# Decrease of IL-1β and TNF in the Spinal Cord Mediates Analgesia Produced by Ankle Joint Mobilization in Complete Freund Adjuvant-Induced Inflammation Mice Model

**DOI:** 10.3389/fphys.2021.816624

**Published:** 2022-01-14

**Authors:** Carlos Minoru Omura, Daniela Dero Lüdtke, Verônica Vargas Horewicz, Paula Franson Fernandes, Taynah de Oliveira Galassi, Afonso Shiguemi Inoue Salgado, Juliete Palandi, Heloiza dos Santos Baldança, Edsel B. Bittencourt, Josiel Mileno Mack, Lynsey A. Seim, Daniel Fernandes Martins, Franciane Bobinski

**Affiliations:** ^1^Experimental Neuroscience Laboratory (LaNEx), Graduate Program in Health Sciences, University of Southern Santa Catarina, Palhoça, Brazil; ^2^Faculty of Physical Therapy, University of Southern Santa Catarina, Palhoça, Brazil; ^3^Natural Quanta Wellness Center, Windermere, FL, United States; ^4^Laboratory of Experimentation in Neuropathology (LEN), Graduate Program in Neuroscience, Department of Biochemistry, Federal University of Santa Catarina, Florianopolis, Brazil; ^5^Coastal Health Institute, Jacksonville, FL, United States; ^6^Graduate Program in Medical Sciences, Department of Medical Clinic, Federal University of Santa Catarina (UFSC), Florianopolis, Brazil; ^7^Faculty of Medicine, University of Southern Santa Catarina, Palhoça, Brazil; ^8^Department of Hospital Internal Medicine, Mayo Clinic, Jacksonville, FL, United States

**Keywords:** joint mobilization, manual therapy, neuroimmunomodulation, inflammatory cytokines, macrophages

## Abstract

**Objective:**

This study aims to investigate the effects of ankle joint mobilization (AJM) on mechanical hyperalgesia and peripheral and central inflammatory biomarkers after intraplantar (i.pl.) Complete Freund’s Adjuvant (CFA)-induced inflammation.

**Methods:**

Male Swiss mice were randomly assigned to 3 groups (*n* = 7): Saline/Sham, CFA/Sham, and CFA/AJM. Five AJM sessions were carried out at 6, 24, 48, 72, and 96 h after CFA injection. von Frey test was used to assess mechanical hyperalgesia. Tissues from paw skin, paw muscle and spinal cord were collected to measure pro-inflammatory (TNF, IL-1β) and anti-inflammatory cytokines (IL-4, IL-10, and TGF-β1) by ELISA. The macrophage phenotype at the inflammation site was evaluated by Western blotting assay using the Nitric Oxide Synthase 2 (NOS 2) and Arginase-1 immunocontent to identify M1 and M2 macrophages, respectively.

**Results:**

Our results confirm a consistent analgesic effect of AJM following the second treatment session. AJM did not change cytokines levels at the inflammatory site, although it promoted a reduction in M2 macrophages. Also, there was a reduction in the levels of pro-inflammatory cytokines IL-1β and TNF in the spinal cord.

**Conclusion:**

Taken together, the results confirm the anti-hyperalgesic effect of AJM and suggest a central neuroimmunomodulatory effect in a model of persistent inflammation targeting the pro-inflammatory cytokines IL-1β and TNF.

## Introduction

The inflammatory process is an essential mechanism of the immune system that allows the body to remove harmful stimuli and recover damaged tissue, restoring tissue homeostasis (Medzhitov, 2008; Ahmed, 2011). However, the persistence of harmful stimulus and the impaired resolution of inflammation forms the basis of many chronic inflammatory diseases, including cardiovascular disease, diabetes, certain cancers and bowel, arthritis, and osteoporosis (Libby, 2007). In this way, inflammation is no longer a protective and specific response of the organism and can generate disorders such as chronic pain, sleep disorders, anxiety, and depression. These comorbidities significantly reduce the individual’s quality of life, also impairing their productive capacity and burdening public health and socioeconomic systems (Institute of Medicine (US) Committee on Advancing Pain Research, Care, and Education, 2011). Experimental studies in animals contribute to the elucidation of the pathological mechanisms underlying inflammatory conditions. Complete Freund Adjuvant (CFA) is a bacterial antigens-emulsifier routinely used to study inflammatory pain, as it mimics inflammatory pathologies, such as rheumatoid arthritis, in addition to presenting high reproducibility in rats and mice (Latremoliere and Woolf, 2009; Fehrenbacher et al., 2012; Ananthi et al., 2017; Montilla-García et al., 2017; Sung et al., 2017; McCarson and Fehrenbacher, 2021). Rheumatoid arthritis is the most common form of inflammatory arthritis and is among the most prevalent joint diseases in the world (1% of the population), being one of the main causes of reduced quality of life (Alamanos and Drosos, 2005). This disease affects middle-aged people, young people, and especially the elderly, causing pain and severe physical disability (Woolf and Pfleger, 2003; Smolen et al., 2016; Perretti et al., 2017).

CFA contains lipoproteins, glycolipids, and peptidoglycans that are recognized by antigen-presenting cells through pattern recognition receptors (PRRs), such as Toll-like receptors (TLRs) 1, 2, 4, and 6 (Zhao et al., 2015), nucleotide-binding oligomerization domain-containing protein 2 (NOD2) and beta-glucan receptor dectin-1. PRRs activate intracellular mechanisms such as the nuclear factor-kB (NF-kB) and inflammasome transcription pathways, which regulate multiple genes, including those that control the inflammatory response, producing and releasing cytokines as the tumor necrosis factor (TNF) and interleukins (IL)-1β and IL-6 (Underhill et al., 1999; Means et al., 2001; Tapping and Tobias, 2003; Ferwerda et al., 2005; Yadav and Schorey, 2006; Kleinnijenhuis et al., 2009). These inflammatory mediators recruit neutrophils, reaching their peak around 24 h after the injection of CFA. Over time, the cell profile changes, grouping hematogenous monocytes and macrophages, which become dominant at the inflammation site 72–96 h after CFA injection (Ghasemlou et al., 2015).

The phenotype expressed in these cells can resolve or intensify the maintenance of inflammation in the microenvironment, making it chronic. Initially, monocytes differentiate into M1 macrophages, releasing pro-inflammatory mediators in order to activate and recruit additional immune cells to contain the invading agent. However, at a later stage, the macrophages’ phenotype changes to the M2 profile, which releases anti-inflammatory mediators such as IL-4, IL-10, and transforming growth factor-β (TGF-β), which contribute to the inflammation resolution and tissue remodeling (Mantovani et al., 2004; Ambarus et al., 2012; Jaguin et al., 2013; Ghasemlou et al., 2015).

CFA induces peripheral and central sensitization of nociceptive neurons that can lead to painful conditions such as hyperalgesia and allodynia to thermal and mechanical stimuli (Raghavendra et al., 2004; Ghasemlou et al., 2015; Zucoloto et al., 2019). Peripheral inflammation has been shown to produce robust microglia reactivity that precedes the astrocytes activation in the spinal cord. These cells increase expression of pro-inflammatory cytokines such as TNF, IL-1β, and IL-6 in parallel with the onset of peripheral inflammation-induced pain (4 h) and are responsible for maintaining pain facilitation (up to 14 days) after i.pl. administration of CFA (Raghavendra et al., 2004; Ghasemlou et al., 2015; Zucoloto et al., 2019).

Our research group demonstrated that ankle joint mobilization (AJM) could reduce plantar surgical incision and complex regional pain syndrome type I (CRPS-I) related nociception in mice (Martins et al., 2012, 2013b; Salgado et al., 2019), and neuropathic pain after peripheral nerve injury in rats (Martins et al., 2011). Other preclinical studies demonstrated that joint mobilization (JM) technique promotes gain of movement and decreased pain (Sluka and Wright, 2001; Sluka et al., 2006; Nielsen et al., 2009). Among the various forms of application observed in clinical practice and tested in experimental studies, the passive ankle oscillatory JM was used in this study, consisting of low-speed rhythmic movements across the physiological range of motion. This technique has been shown to produce analgesic effects in both humans and animal models (Sluka and Wright, 2001; Sluka et al., 2006; Martins et al., 2011, 2012; Salgado et al., 2019).

Systematic reviews of clinical studies demonstrate the effects of manual therapy with JM for the treatment of plantar heel pain (Mischke et al., 2017), patellofemoral pain syndrome (Jayaseelan et al., 2018), and ankle sprains (Weerasekara et al., 2018). In addition, Alonso-Perez et al. (2017) compared three manual therapy techniques on the pressure pain threshold in 75 healthy individuals and observed that JM induced a greater hypoalgesic effect compared to high velocity low amplitude technique and cervical lateral glide mobilization.

Although the analgesic effect and part of the neurophysiological mechanisms of JM have been demonstrated in other animal models of pain, they have not been investigated in a model of CFA-induced persistent inflammatory pain. Thus, we aim to evaluate the anti-hyperalgesic effect and the peripheral and central anti-inflammatory mechanism in a model of i.pl. CFA-induced inflammation in mice. The pro- and anti-inflammatory cytokines levels in the inflamed paw and spinal cord, as well as the macrophage phenotype present at the inflammation site were measured. Thus, this investigation can serve as a basis for future clinical studies that evaluate the effects of JM on the pathogenesis of chronic inflammatory diseases and their comorbidities, as well as for understanding the JM mechanisms and support the best clinical approach.

## Materials and Methods

### Animals

All experimental protocols were approved by the Ethics Committee on Animal Use (CEUA-UNISUL, protocol n. 19.003.4.01.IV). Experiments were carried out using male Swiss mice (60 days old, 35–45 g), obtained from the animal facility of the Federal University of Santa Catarina (Florianopolis, Brazil). The animals were housed in the Experimental Neuroscience Laboratory (LaNEx) of the University of Southern Santa Catarina, in a room maintained at 22 ± 2°C, on a 12 h-light/dark cycle (lights on at 06:00 a.m.), with free access to food and water.

The number of animals per group was determined using the sample size equation without replacement (Wayne and Chad, 2018), using a 95% confidence interval: *N* = {[(z alpha + z beta) * s]/sigma}^2^. Thus, applying the values to the formula: *N* = {[(1.96 + 1.28) * 30]/40}^2^ = 5.9. A percentage of 15% was added to the sample N due to the error inherent in biological experiments and sample loss. Thus, each group was composed of 7 animals. The animals were randomly grouped. For this, the allocation sequence was generated by a lot. A blinded experimenter randomly distributed the animals among the three experimental groups: Saline/Sham, animals injected with 0.9% NaCl (20 μl, i.pl.) and isoflurane anesthesia (Isoforine^®^, Cristália Prod. Quím. Farm. Ltda., São Paulo, SP, Brazil) for the same time as AJM; CFA/Sham, animals injected with 70% CFA (20 μl, i.pl.) and isoflurane anesthesia for the same time as AJM; CFA/AJM, animals injected with 70% CFA (20 μl, i.pl.) and treated with AJM under isoflurane anesthesia for 9 min. The animals were randomly housed at a maximum number of 15 per polypropylene cage (49 × 34 × 16 cm) with stainless steel grids with wood shavings.

The experiments were carried out under the ethical principles established by the Brazilian College of Animal Experimentation (COBEA) and the legislation for the protection of animals used for scientific purposes (Directive 2010/63/EU revising Directive 86/609/EEC) and the National Institutes of Health Guidelines.

### Study Design

**FIGURE 1:**
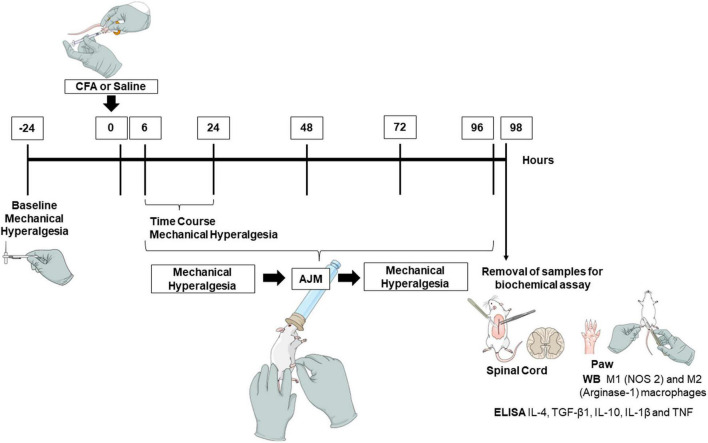
Experimental design. To conduct this study, we performed baseline assessments to determine the parameters of mechanical hyperalgesia using the von Frey test. From this, we induced the inflammatory pain model by injection of CFA or saline (i.pl.), and performed the evaluations from 6, 24, 48, 72, and 96 h, pre- and post-treatments (AJM or Sham). Approximately 98 h after the injection of CFA and 2 h after the last treatment with AJM, mice were anesthetized and euthanized to remove the samples (spinal cord and paw) for biochemical assay. CFA, Complete Freund’s Adjuvant; AJM, Ankle Joint Mobilization; ELISA, Enzyme-Linked Immunosorbent Assay; TNF, Tumor Necrosis Factor; IL, Interleukin; WB, Western blotting; NOS 2, Nitric Oxide Synthase 2; TGF-β1, Transforming Growth Factor-β.

Initially, baseline parameters of mechanical hyperalgesia were determined using the von Frey test, followed by the CFA-induced inflammation model (i.pl.) or saline (i.pl.), used as a control. The assessments of mechanical hyperalgesia were performed prior to, and 6, 24, 48, 72, and 96 h after the interventions (AJM or Sham). 98 h after induction of the CFA model and about 2 h after the last treatment with AJM the mice were anesthetized, and samples collected for Enzyme-Linked Immunosorbent Assay (ELISA) and Western blotting (Figure 1). All evaluations were performed by blinded researchers regarding the animal’s group allocation treatment. This study followed the ARRIVE guideline (Animals in Research: Reporting *In vivo* Experiments) (Percie du Sert et al., 2020). In order to verify the reproducibility of the data presented in this article, the experiments were replicated in two independent sets.

### Complete Freund’s Adjuvant-Induced Paw Inflammation Model

The model of CFA-induced peripheral inflammatory pain generates a state of persistent inflammation (Ghasemlou et al., 2015). In this sense, the animals were randomly anesthetized (1–2% isoflurane at 100% oxygen). After checking the unconsciousness state, the mice received the i.pl. CFA (70%, 20 μl, Sigma-Aldrich, St. Louis, MO, United States) or saline (NaCl 0.9%, 20 μl, control group) on the right hind paw plantar surface. The animals were under observation until the return of consciousness. After, they remained in their housing cage in the laboratory’s experiment room with free access to food and water for approximately 5 h, until the beginning of the behavioral evaluations.

### Ankle Joint Mobilization

AJM was performed on the right hind paw, keeping the knee joint flexed and performing rhythmic and slow movements of flexion and extension of the ankle joint, respecting the range of joint movement (as shown in Figure 1). As described before (Martins et al., 2012), slow AJM was performed with a frequency of approximately 40 movements per minute and controlled with a metronome. Five AJM sessions were carried out at 6, 24, 48, 72, and 96 h after CFA injection. The mobilizations occurred for 9 min and divided into 3 periods of 3 min between each period, with a 30-s interval between each cycle. All animals were anesthetized with 1–2% isoflurane at 100% oxygen throughout the procedure of AJM or Sham (Martins et al., 2012). The Sham procedure consisted of positioning the experimenter’s hands to keep the hip, knee and ankle joints flexed, without performing moving, during the same time period as the AJM. The two researchers who performed the treatment were blinded regarding the experimental group.

### Mechanical Hyperalgesia

The evaluation of mechanical hyperalgesia was carried out using a von Frey monofilament of 0.6 g (VFH, Stoelting, IL, United States). The animals were acclimated in individual acrylic chambers (9 × 7 × 11 cm) on a wire mesh to allow the application of the monofilament on the paw ventral surface. The tests were randomly performed among the animals in each experimental group. The number of paw withdrawals was recorded and expressed as a percentage of the withdrawal response. The monofilament application was perpendicular to the plantar surface and performed 10 times. Sufficient pressure must be applied to provide a curvature of the filament and the lifting of the right hind paw, which is considered a positive response (Rosas et al., 2018).

### Biochemical Assays

Approximately 98 h after administration of CFA or saline, and 2 h after the last treatment with AJM, the mice were anesthetized (1–2% isoflurane at 100% oxygen) and euthanized by decapitation for the removal of the right hind paw skin and muscle and spinal cord (L4-L6). After dissection, the samples were immediately frozen in liquid nitrogen and stored in a –80°C freezer until the analysis.

### Enzyme-Linked Immunosorbent Assay

Spinal cord and paw samples were homogenized in Ultra-Turrax Homogenizer (T-18, IKA Works, Wilmington, NC, United States) with phosphate-buffered saline (PBS) containing Tween 20 (0.05%), PMSF (0.1 mM), EDTA (10 mM), aprotinin (2 ng/mL) and benzethonium chloride (0.1 mM). Afterward, the samples were centrifuged at 6,000 × g for 15 min (at 4°C) and the supernatant collected and stored at –80°C. The supernatant total protein content was measured by the Bradford method, using a standard calibration curve with BSA (0.05–0.5 mg/mL). Aliquots with 100 μl were used to measure cytokine concentrations (IL-10, IL-4, TGF-β1, IL-1β, and TNF) by ELISA kits for mice (Invitrogen/Thermo Fisher Scientific, Waltham, MA), according to the manufacturer’s instructions.

Cytokine concentrations were measured by interpolating a 7-point standard curve with colorimetric assays reading at 450 nm on a plate spectrophotometer (Perlong DNM-9602, Nanjing Perlove Medical Equipment Co., Nanjing, China). For correction of optical imperfections in the plate, the reading at 540 nm was subtracted from the reading at 450 nm. The values were expressed as pg (cytokine) per mg (protein).

### Western Blotting

The Western blotting assay was used to determine proteins immunocontent: NOS 2, an M1 macrophage marker, and Arginase-1, an M2 macrophage marker. Samples were homogenized and incubated in RIPA lysis buffer (composed of 1% Nonidet P-40, 0.5% sodium deoxycholate, 0.1% SDS and PBS) plus 100 mM sodium orthovanadate, 100 mM PMSF and cocktail of 1% protease inhibitors (Sigma-Aldrich, St. Louis, MO, United States). Then, the samples were incubated on ice for 30 min. After centrifugation at 6,000 × g for 20 min (at 4°C), the supernatant was collected, separated, and stored in a –80°C freezer. Protein content was measured according to the Bradford method, using a standard calibration curve with BSA (0.05–0.5 mg/mL). Total protein aliquots (50 μg) were boiled at 95°C for 5 min in 25% volume in Laemmli buffer (1 M sodium phosphate pH 7.0, 10% sodium dodecyl sulfate (SDS), 10% β-mercaptoethanol, 50% glycerol, 0.1% bromophenol blue).

Samples were submitted to electrophoresis on 8% polyacrylamide gel. Proteins were then transferred onto PVDF membrane for 2 h at 90 V constant voltage. The membrane was incubated for 1 h in blocking solution (5% milk powder Molico^®^) and then incubated overnight at 4°C with the following primary antibodies: Rabbit anti-iNOS (or NOS 2) (NBP1-33780, Novus Biologicals, Centennial, CO, United States); Rabbit anti-Arginase-1 (93668S, Cell Signaling Technology, Danvers, MA, United States) or Rabbit anti-β-actin-HRP (Sigma-Aldrich, St. Louis, MO, United States). After washing in Tris Buffer Saline with Tween^®^ 20 (TBS-T) (137 mM NaCl and 20 mM Tris HCl + 0.1% Tween^®^ 20, pH 7.6), the membranes were incubated with the specific secondary antibody (Abcam, Cambridge, United Kingdom) conjugated to peroxidase at room temperature for 1 h. After this period, a new 30-min wash with TBS-T was performed, followed by exposure of the membranes for 1 min to the chemiluminescence kit (ECL) and detection using an imaging system (iBright Imaging Systems, Invitrogen/Thermo Fisher Scientific, Waltham, MA, United States). The quantification of protein bands was performed by densitometry using the iBright Analysis Software (Invitrogen/Thermo Fisher Scientific, Waltham, MA, United States). The values were normalized according to the β-actin values and graphically expressed as arbitrary units in relation to the control (Saline/Sham).

### Statistical Analysis

Data were analyzed using the GraphPad Prism^®^ software version 8.0 (La Jolla, California, United States). The normal distribution was verified using the Shapiro-Wilk test, and the parametric results presented as mean ± standard deviation (SD). Mechanical hyperalgesia data were compared using two-way analysis of variance (ANOVA), followed by Tukey’s *post hoc* test for multiple comparisons. For biochemical data, one-way ANOVA followed by Tukey’s *post hoc* test was used. Values were considered statistically significant when *p* < 0.05.

## Results

### Ankle Joint Mobilization Decreases Complete Freund’s Adjuvant-Induced Mechanical Hyperalgesia

**FIGURE 2:**
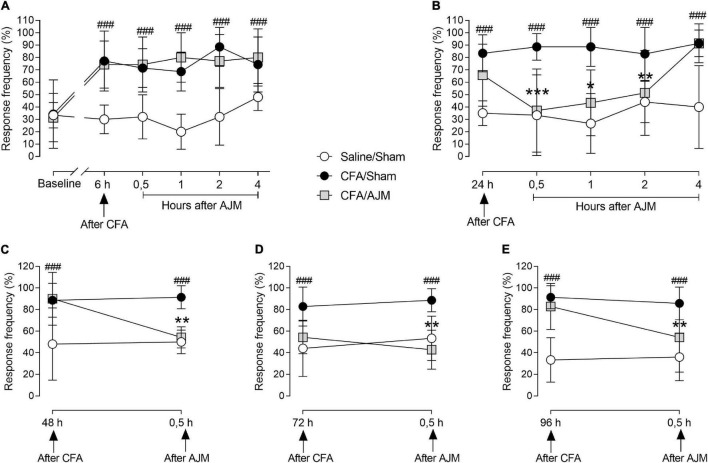
Evaluation of mechanical hyperalgesia performed by the von Frey test, in male Swiss mice submitted to the intraplantar CFA model and treated with AJM. Baseline evaluations (prior to the procedure) are presented: the measures at 6 **(A)** and 24 h **(B)** after intraplantar CFA and the periods of 0.5, 1, 2, and 4 h **(A,B)** after AJM. Also, 48 **(C)**, 72 **(D)**, and 96 h **(E)** after injection of CFA and 0.5 h after AJM. The values represent the mean ± SD of seven animals per group, statistically compared by two-way ANOVA with repeated measures followed by Tukey’s *post-hoc* test. ^###^*p* < 0.001, when compared to the Saline/Sham group; *p < 0.05, **p < 0.01, and ***p < 0.001, when compared to the CFA/Sham group. CFA, Complete Freund’s Adjuvant; AJM, Ankle Joint Mobilization.

The i.pl. CFA injection produced intense hyperalgesia in the paw of animals of the CFA/Sham group, with an increased response to the mechanical stimulus evaluated by the von Frey test up to 96 h after CFA administration, when compared to the Saline/Sham group (*p* < 0.001, Figure 2). Figure 2A highlights the time course of mechanical hyperalgesia 6 h after intraplantar injection of CFA and 0.5, 1, 2, and 4 h after the first treatment with AJM. Mechanical hyperalgesia was not reduced by the first AJM treatment when comparing to the CFA/Sham and CFA/AJM groups.

Figure 2B shows the time course of mechanical hyperalgesia 24 h after the i.pl. CFA-induced inflammation and 0.5, 1, 2, and 4 h after the second treatment with AJM. Mechanical hyperalgesia was observed for 24 h and in the subsequent periods evaluated in the CFA/Sham group when compared to the Saline/Sham group (*p* < 0.001). AJM reduced hyperalgesia at 0.5 (*p* < 0.001), 1 (*p* < 0.01), and 2 h (*p* < 0.05) following treatment in the CFA/AJM group when compared to CFA/Sham, returning to baseline values in the 4th hour after AJM.

The evaluation of mechanical hyperalgesia was performed prior to daily treatment and 0.5 h after AJM on the 2nd (48 h), 3rd (72 h), and 4th day (96 h) after the i.pl. administration of CFA (Figures 2C–E, respectively). Mechanical hyperalgesia was maintained from 48 to 96 h and in all periods later evaluated in the CFA/Sham group when compared to the Saline/Sham group (*p* < 0.001). The AJM significantly evoked anti-hyperalgesic effects when compared to animals in the CFA/Sham group 48 (*p* < 0.01, Figure 2C), 72 (*p* < 0.001, Figure 2D) and 96 h (*p* < 0.05, Figure 2E) after i.pl. CFA administration.

### Ankle Joint Mobilization Does Not Alter the Concentration of Anti-inflammatory (IL-4, TGF-β1, and IL-10) and Pro-inflammatory (IL-1β and TNF) Cytokines After i.pl. Complete Freund’s Adjuvant

**FIGURE 3:**
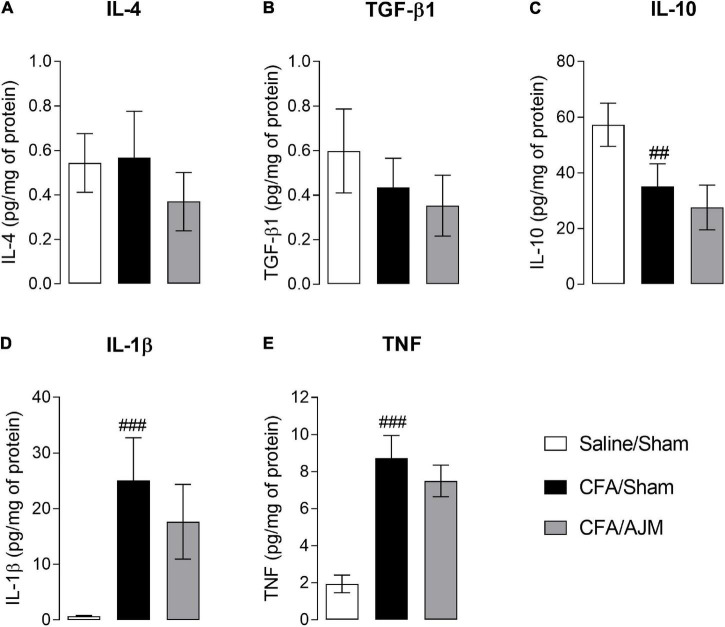
Effects of AJM on the concentration of anti-inflammatory IL-4 **(A)**, TGF-β1 **(B)** and IL-10 **(C)**, and pro-inflammatory IL-1β **(D)** and TNF **(E)** cytokines on the paw of male Swiss mice 98 h after intraplantar CFA-induced inflammation. Data are expressed as mean ± SD of 7 animals per group, statistically assessed by one-way ANOVA followed by Tukey’s *post hoc* test. ^##^*p* < 0.01 and ^###^*p* < 0.001 when compared to the Saline/Sham group. CFA, Complete Freund’s Adjuvant; AJM, Ankle Joint Mobilization.

Figure 3 shows the IL-4, TGF-β1, IL-10, IL-1β, and TNF levels in the paw tissue 98 h after i.pl. CFA administration. The injection of CFA and AJM did not significantly alter the IL-4 (Figure 3A) and TGF-β1 levels (Figure 3B) when compared Saline/Sham vs. CFA/Sham, and CFA/Sham vs. CFA/AJM. However, i.pl. injection of CFA reduced the concentrations of the anti-inflammatory cytokine IL-10 (*p* < 0.01, Figure 3C) and increased the paw concentrations of the pro-inflammatory cytokines IL-1β (*p* < 0.001, Figure 3D) and TNF (*p* < 0.001, Figure 3E) in the CFA/Sham mice when compared to the Saline/Sham group. Although lower concentrations of these pro-inflammatory cytokines have been observed in the CFA/AJM group (especially regarding IL-1β) compared to the CFA/Sham group, this reduction was not statistically significant.

### Ankle Joint Mobilization Decreases Arginase-1 Immunocontent After i.pl. Complete Freund’s Adjuvant

**FIGURE 4:**
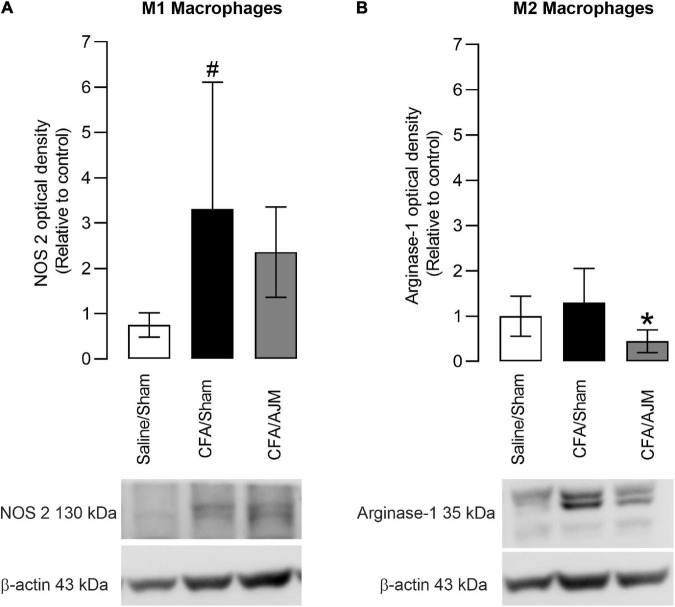
Effects of AJM on the immunocontent of M1 (NOS 2) **(A)** and M2 (Arginase-1) **(B)** macrophage markers in the paw of male Swiss mice 98 h after intraplantar CFA-induced inflammation. Data are expressed as mean ± SD of 7 animals per group, statistically assessed by one-way ANOVA followed by Tukey’s *post hoc* test. ^#^*p* < 0.05 when compared to the Saline/Sham group. *p < 0.05, when compared to the CFA/Sham group. CFA, Complete Freund’s Adjuvant; AJM, Ankle Joint Mobilization.

The effects of AJM on the phenotype of pro-inflammatory (M1) or anti-inflammatory (M2) macrophages 98 h after the i.pl. administration of CFA is shown in Figure 4. The i.pl. injection of CFA increased the immunocontent of the M1 marker (NOS 2, *p* < 0.05, Figure 4A), but did not alter the immunocontent of the M2 marker (Arginase-1, *p* = 0.5073, Figure 4B) on the paws of animals in the CFA/Sham group when compared to the Saline/Sham group. AJM did not reduce the NOS 2 immunocontent (*p* = 0.5540, Figure 4A) increased by the CFA, but significantly reduced the Arginase-1 immunocontent (*p* < 0.05, Figure 4B) compared to CFA/Sham group.

### Ankle Joint Mobilization Decreases the Concentration of Pro-inflammatory Cytokines in the Spinal Cord After i.pl. Complete Freund’s Adjuvant

**FIGURE 5:**
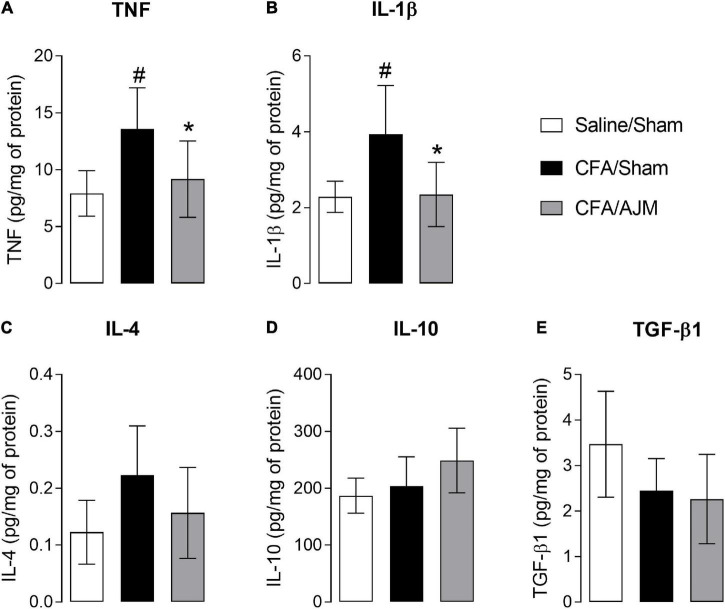
Effects of AJM on the concentrations of pro-inflammatory IL-1β **(A)** and TNF **(B)**, and anti-inflammatory IL-4 **(C)**, IL-10 **(D)** and TGF-β1 **(E)** cytokines in the spinal cord of Swiss male mice 98 h after intraplantar CFA-induced inflammation. Data are expressed as mean ± SD of 7 animals per group, statistically assessed by one-way ANOVA followed by Tukey’s *post hoc* test. ^#^*p* < 0.05 when compared to the Saline/Sham group. *p < 0.05, when compared to the CFA/Sham group. CFA, Complete Freund’s Adjuvant; AJM, Ankle Joint Mobilization.

Figure 5 shows the concentration of TNF, IL-1β, IL-4, IL-10, and TGF-β1 in the spinal cord 98 h after the i.pl. administration of CFA. CFA injection increased TNF (*p* < 0.05, Figure 5A) and IL-1β levels (*p* < 0.05, Figure 5B), but did not alter IL-4, IL- 10, and TGF-β1 levels in the spinal cord of animals from CFA/Sham group when compared to the Saline/Sham group. AJM reduced TNF (*p* < 0.05, Figure 5A) and IL-1β levels (*p* < 0.05, Figure 5B) and did not change IL-4, IL-10, and TGF-β1 levels in the spinal cord of mice in the CFA/AJM group compared to the CFA/Sham group.

## Discussion

The present study demonstrated the anti-hyperalgesic effects elicited by AJM on CFA-induced inflammatory pain. The immunomodulatory mechanism of AJM in the spinal cord and at the inflammation site has been partially elucidated. Accordingly, we showed that AJM reduced the concentrations of pro-inflammatory cytokines IL-1β and TNF in the spinal cord, suggesting a neuroimmunomodulatory effect on central sensitization, but did not alter the cytokine concentrations in the inflamed paw, although it promoted M2 macrophages reduction.

AJM decreased nociceptive behavior after inducing paw inflammation is consistent with previous findings from our laboratory and other research groups regarding the analgesic effects of JM on mechanical hyperalgesia in mice and rats (Sluka and Wright, 2001; Sluka et al., 2006; Moss et al., 2007; Martins et al., 2011, 2012, 2013b). As also demonstrated in the postoperative pain model (Martins et al., 2013b), our results show that the analgesic effects elicited by AJM is evident 24 h after CFA. The maximum mechanical hyperalgesia observed following i.pl. CFA in mice occurred between 6 and 24 h after inflammation induction (Ghasemlou et al., 2015). Likewise, studies showing changes in the cell profile in CFA models highlight initially an intense migration of neutrophils to the injured site, with the inflammatory process persisting within 6 h after CFA (Ghasemlou et al., 2015; Yin and Heit, 2018; Fattori et al., 2019).

Neutrophils are highly effective in combating pathogens. However, in this process, they produce and release reactive oxygen species such as nitric oxide, superoxide anion, and hydrogen peroxide, which are highly toxic to bacteria but cause damage to the tissue (Yin and Heit, 2018). Furthermore, in this initial stage of inflammation, neutrophils release prostaglandins, leukotrienes, and regulate the synthesis of cytokines such as macrophage inflammatory protein (MIP)-α and β, TGF-β, IL-1α, and β, TNF and IL-6 (Kasama et al., 2005; Yin and Heit, 2018). This creates an intense pro-inflammatory and hyperalgesic environment that was not modified by the first treatment with AJM as observed in our study. Also, the literature shows that neutrophils and other innate immune cells secrete many inflammatory mediators that produced pain, but also produce opioid peptides (Plein and Rittner, 2018). Cyclophosphamide immunosuppressor injection promoted an increase in mechanical and thermal hyperalgesia after CFA-induced inflammation supporting a role for immune cells in endogenous tonic analgesia (Sauer et al., 2014). However, Martins and colleagues demonstrated that the administration of fucoidin (a leukocyte migration inhibitor) did not alter the analgesic effects of AJM in the animal model of postoperative pain, suggesting these effects are not dependent of neutrophil-derived opioids at the injury site (Martins et al., 2012). Other skin immune cells such as dendritic and Langerhans cells can cause hypersensitivity by directly interacting with nociceptors. Also, fibroblasts, keratinocytes, muscle cells, and others can be activated and secrete factors that activate or sensitize nociceptors (Roosterman et al., 2006; Radtke et al., 2010; Wilson et al., 2013).

Regarding the mechanism related to CFA-induced inflammation, this is a pioneer investigation concerning the effects of AJM on the central and peripherical modulation of the immune system. It has been shown that CFA and AJM do not significantly alter the concentrations of anti-inflammatory cytokines (IL-4, TGF-β1, and IL-10). Although CFA injection increases the concentrations of pro-inflammatory cytokines (IL-1β and TNF), AJM was not able to reduce them when measured in the mice’s paw. In addition, we demonstrated, for the first time, the phenotypic characteristic of infiltrated macrophages at the inflammation site 98 h after CFA and approximately 2 h after the last treatment with AJM. Monocytes and macrophages are the main inflammatory cells present in the late stage of inflammation (Mosser and Edwards, 2008; Ghasemlou et al., 2015). Macrophages demonstrate a predominant pro- or anti-inflammatory state depending on the balance between the M1 and M2 phenotypes (Gordon and Martinez, 2010). The current study shows that CFA increases the density of M1 macrophages without changing the density of M2 at the inflammation site. Consistent with our findings, Ghasemlou et al. (2015) demonstrated that pro-inflammatory macrophages were the predominant immune cells in the skin from 3 to 14 days after CFA injection. In contrast, in that same period, higher anti-inflammatory profile-Ly6C*^low/med^* macrophages were reduced after the third day of CFA treatment but still increased in relation to naive animals. It is possible that the discrepancy concerning the phenotype of anti-inflammatory cells is due to the high sensitivity of the flow cytometry technique used to quantify immune cell subpopulations in the study of Ghasemlou et al. (2015) in relation to the Western Blotting technique used in our study.

We observed that AJM did not reduce the infiltrated M1 macrophages following CFA administration, while reducing M2 macrophages density. This phenotypic distribution is consistent with the profile of inflammatory cytokines measured in the paw of animals treated with AJM, where low levels of IL-4, TGF-β1, and IL-10 were observed, cytokines secreted by M2 macrophages with anti-inflammatory, pro-resolving and tissue repair properties (Mantovani et al., 2004; Gordon and Martinez, 2010). Although AJM reduced the levels of IL-1β and TNF, cytokines secreted by M1 macrophages with pro-inflammatory properties, this effect was not significant. With these results, we suggest that, in the period evaluated, the animals presented a peak of the pro-inflammatory macrophage phase and the AJM was not able to interfere in the normal course of the M1 macrophage peak. However, AJM would likely have a better chance of intensifying the switch from the M1 to M2 macrophage phenotype at a transition point between the two phases, rather than at the peak of M1 participation. Further studies are necessary to study the potential alteration of cytokines production and M1/M2 polarization of macrophages by AJM at other time points along the course of inflammation and not only at one time-point, to understand when this phenotype change occurs.

Surprisingly, AJM induced neuroimmunomodulatory effects at the spinal cord, reducing the concentrations of IL-1β and TNF that were increased after CFA administration. Evidence from the literature indicates that neuroinflammation plays an essential role in the nociceptive transmission at the spinal and supraspinal levels. Recent studies indicate that the activation of glial cells with the subsequent release of inflammatory mediators critically contributes to the central sensitization in the pathogenesis of inflammatory pain, especially in CFA-induced pain (Raghavendra et al., 2004; Zhao et al., 2015; Zucoloto et al., 2019). Moreover, it is well established that the increase in glutamatergic transmission, both in the periphery and spinal cord, is one of the typical pathophysiological characteristics of pain (Du et al., 2003; Osikowicz et al., 2013). Peripheral CFA injection induces persistent nociceptive stimuli with an intense glutamatergic release, characterized by rapid activation of the microglia and astrocytes in the spinal cord (Du et al., 2003; McMahon and Malcangio, 2009). These cells release bioactive molecules such as the neuromodulators TNF and IL-1β, amplifying the intensity and duration of pain (Samad et al., 2001; Narita et al., 2008). Previous studies have shown that inflammatory factors such as IL-1β, IL-6, and TNF mediate pain signaling by direct sensitization of nociceptive neurons and by triggering the production of other hyperalgesic mediators (Samad et al., 2001; Ligthart et al., 2016). Consistent with these findings, IL-1 receptor 1 (IL-1R1) and TNF receptor 1 (TNFR1) have been identified in neurons of the spinal cord dorsal horn and primary afferent terminals (Holmes et al., 2004; Zhang et al., 2008). In parallel, cytokines such as IL-4 and IL-10 activate glial cells in a way to create an endogenous anti-inflammatory and neuroprotective environment, promoting neuronal survival and reversing hyperalgesia in animal models of chronic pain (Milligan and Watkins, 2009). To reinforce our findings, the CFA i.pl. injection increased the protein concentrations and mRNA of the pro-inflammatory cytokines IL-1β, IL-6, and TNF in the spinal cord of rats, observed from 4 h to 14 days (Raghavendra et al., 2004), and in mice observed at 3 day following CFA administration (Zucoloto et al., 2019). The expression of IL-1R1 on the membrane surface of neurons and astrocytes in the spinal cord, but not in microglia, has also been demonstrated in mice submitted to CFA i.pl. injection (Holló et al., 2017).

Previously, it was demonstrated that peripheral joint mobilization could reduce the activation of pain-related areas in the spinal cord of rats (Malisza et al., 2003). A study with functional magnetic resonance showed a reduction of activated areas in the lumbar spinal cord of rats submitted to intra-articular injection of capsaicin in the right ankle and treated with AJM for 9 min (Malisza et al., 2003). Our research group has previously shown that the neurobiological mechanism underlying the analgesic effects of AJM in mice involves the activation of opioids (Martins et al., 2012), adenosine (Martins et al., 2013b), and cannabinoids receptors (Martins et al., 2013a) in the spinal cord. Interestingly, these receptors are coupled to an inhibitory G (Gi) protein (G-PCR). It is known that G-PCRs regulate the ion channels, which play an essential role in neuronal function through the regulation of electrical currents and ion concentrations across the cell membrane (Xiang et al., 2019). In addition, G-PCR agonists inhibit pain transmission by blocking voltage-gated Ca^2+^ channels, reducing the release of excitatory neurotransmitters from presynaptic terminals of nociceptive sensory neurons and hyperpolarizing second-order neurons by activating inward-rectifier potassium channels (Kohno et al., 1999; Pan et al., 2002). The cannabinoids, opioids, and adenosine receptors are located in the peripheral sensory nerve endings (Agarwal et al., 2007; Lima et al., 2010; Stein and Machelska, 2011) and the superficial layers of the spinal cord dorsal horn (Pettersson et al., 2002; Wang et al., 2007; Zhang et al., 2008).

Supporting the hypothesis that AJM can inhibit glial cells in the spinal cord, our research group demonstrated AJM performed for 15 days after rat sciatic nerve crushing reduces mechanical hyperalgesia and GFAP and CD11 b/c immunoreactivity in the spinal cord, astrocyte and microglia activation markers, respectively (Martins et al., 2011). In this context, although we have not observed a significant change in the IL-4 and IL-10 levels in the spinal cord, previous results from our research group support that the activation of opioids, adenosine, or cannabinoids receptors may be targeted by AJM-induced endogenous substances, reducing neuronal and glial activity, the release of glutamate and consequently the concentrations of TNF and IL-1β in the spinal cord.

## Conclusion

In conclusion, our results confirm the analgesic efficacy of AJM, in addition to showing a reduction in the concentration of pro-inflammatory cytokines in the spinal cord. Understanding the mechanisms involved in JM therapy can lead to new therapeutic targets that not only may improve pain in patients with inflammatory pathologies but also modify the underlying pathological process. Further, these studies will support clinicians and patients in adhering to physical therapies such as JM as adjuvants to pharmacological treatment. However, future studies are needed to further clarify the underlying mechanisms of JM in the treatment of different pathologies.

## Data Availability Statement

The raw data supporting the conclusions of this article will be made available by the authors, without undue reservation.

## Ethics Statement

The animal study was reviewed and approved by the Ethics Committee on Animal Use from University of Southern Santa Catarina (CEUA-UNISUL, protocol n. 19.003.4.01.IV).

## Author Contributions

FB and DFM: study conception and design. CMO, DDL, VVH, PFF, TOG, JP, and HSB: acquisition of data. FB, JMM, and DFM: analysis and interpretation of data. CMO, FB, LAS, JMM, and DFM: drafting of manuscript. FB, DFM, EBB, and ASIS: critical revision. All authors contributed to the article and approved the submitted version.

## Conflict of Interest

The authors declare that the research was conducted in the absence of any commercial or financial relationships that could be construed as a potential conflict of interest.

## Publisher’s Note

All claims expressed in this article are solely those of the authors and do not necessarily represent those of their affiliated organizations, or those of the publisher, the editors and the reviewers. Any product that may be evaluated in this article, or claim that may be made by its manufacturer, is not guaranteed or endorsed by the publisher.

## References

[B1] Agarwal N. Pacher P. Tegeder I. Amaya F. Constantin C. E. Brenner G. J. (2007). Cannabinoids mediate analgesia largely via peripheral type 1 cannabinoid receptors in nociceptors. *Nat. Neurosci.* 10 870–879. 10.1038/nn1916 17558404 PMC2234438

[B2] Ahmed A. U. (2011). An overview of inflammation: mechanism and consequences. *Front. Biol. (Beijing)* 6:274. 10.1007/s11515-011-1123-9

[B3] Alamanos Y. Drosos A. A. (2005). Epidemiology of adult rheumatoid arthritis. *Autoimmun. Rev.* 4 130–136. 10.1016/j.autrev.2004.09.002 15823498

[B4] Alonso-Perez J. L. Lopez-Lopez A. La Touche R. Lerma-Lara S. Suarez E. Rojas J. (2017). Hypoalgesic effects of three different manual therapy techniques on cervical spine and psychological interaction: a randomized clinical trial. *J. Bodyw. Mov. Ther.* 21 798–803. 10.1016/j.jbmt.2016.12.005 29037630

[B5] Ambarus C. A. Krausz S. van Eijk M. Hamann J. Radstake T. R. Reedquist K. A. (2012). Systematic validation of specific phenotypic markers for in vitro polarized human macrophages. *J. Immunol. Methods* 375 196–206. 10.1016/j.jim.2011.10.013 22075274

[B6] Ananthi S. Gayathri V. Malarvizhi R. Bhardwaj M. Vasanthi H. R. (2017). Anti-arthritic potential of marine macroalgae Turbinaria ornata in Complete Freund’s Adjuvant induced rats. *Exp. Toxicol. Pathol.* 69 672–680. 10.1016/j.etp.2017.06.006 28684087

[B7] Du J. Zhou S. Coggeshall R. E. Carlton S. M. (2003). N-methyl-D-aspartate-induced excitation and sensitization of normal and inflamed nociceptors. *Neuroscience* 118 547–562. 10.1016/s0306-4522(03)00009-5 12699789

[B8] Fattori V. Pinho-Ribeiro F. A. Staurengo-Ferrari L. Borghi S. M. Rossaneis A. C. Casagrande R. (2019). The specialised pro-resolving lipid mediator maresin 1 reduces inflammatory pain with a long-lasting analgesic effect. *Br. J. Pharmacol.* 176 1728–1744. 10.1111/bph.14647 30830967 PMC6514290

[B9] Fehrenbacher J. C. Vasko M. R. Duarte D. B. (2012). Models of inflammation: carrageenan- or complete Freund’s Adjuvant (CFA)-induced edema and hypersensitivity in the rat. *Curr. Protoc. Pharmacol.* Chapter 5, Unit5.4. 10.1002/0471141755.ph0504s56 22382999 PMC4683998

[B10] Ferwerda G. Girardin S. E. Kullberg B.-J. Le Bourhis L. de Jong D. J. Langenberg D. M. (2005). NOD2 and toll-like receptors are nonredundant recognition systems of Mycobacterium tuberculosis. *PLoS Pathog.* 1 279–285. 10.1371/journal.ppat.0010034 16322770 PMC1291354

[B11] Ghasemlou N. Chiu I. M. Julien J.-P. Woolf C. J. (2015). CD11b+Ly6G- myeloid cells mediate mechanical inflammatory pain hypersensitivity. *Proc. Natl. Acad. Sci. U. S. A.* 112 E6808–E6817. 10.1073/pnas.1501372112 26598697 PMC4679057

[B12] Gordon S. Martinez F. O. (2010). Alternative activation of macrophages: mechanism and functions. *Immunity* 32 593–604. 10.1016/j.immuni.2010.05.007 20510870

[B13] Holló K. Ducza L. Hegyi Z. Dócs K. Hegedûs K. Bakk E. (2017). Interleukin-1 receptor type 1 is overexpressed in neurons but not in glial cells within the rat superficial spinal dorsal horn in complete Freund adjuvant-induced inflammatory pain. *J. Neuroinflammation* 14:125. 10.1186/s12974-017-0902-x 28645297 PMC5482961

[B14] Holmes G. M. Hebert S. L. Rogers R. C. Hermann G. E. (2004). Immunocytochemical localization of TNF type 1 and type 2 receptors in the rat spinal cord. *Brain Res.* 1025 210–219. 10.1016/j.brainres.2004.08.020 15464762

[B15] Institute of Medicine (US) Committee on Advancing Pain Research, Care, and Education (2011). *Relieving Pain In America: A Blueprint For Transforming Prevention, Care, Education, And Research.* Washington: The National Academies Press, 10.17226/13172 22553896

[B16] Jaguin M. Houlbert N. Fardel O. Lecureur V. (2013). Polarization profiles of human M-CSF-generated macrophages and comparison of M1-markers in classically activated macrophages from GM-CSF and M-CSF origin. *Cell. Immunol.* 281 51–61. 10.1016/j.cellimm.2013.01.010 23454681

[B17] Jayaseelan D. J. Scalzitti D. A. Palmer G. Immerman A. Courtney C. A. (2018). The effects of joint mobilization on individuals with patellofemoral pain: a systematic review. *Clin. Rehabil.* 32 722–733. 10.1177/0269215517753971 29327606

[B18] Kasama T. Miwa Y. Isozaki T. Odai T. Adachi M. Kunkel S. L. (2005). Neutrophil-derived cytokines: potential therapeutic targets in inflammation. *Curr. Drug Targets. Inflamm. Allergy* 4 273–279. 10.2174/1568010054022114 16101533

[B19] Kleinnijenhuis J. Joosten L. A. van de Veerdonk F. L. Savage N. van Crevel R. Kullberg B. J. (2009). Transcriptional and inflammasome-mediated pathways for the induction of IL-1β production by Mycobacterium tuberculosis. *Eur. J. Immunol.* 39 1914–1922. 10.1002/eji.200839115 19544485

[B20] Kohno T. Kumamoto E. Higashi H. Shimoji K. Yoshimura M. (1999). Actions of opioids on excitatory and inhibitory transmission in substantia gelatinosa of adult rat spinal cord. *J. Physiol.* 518 803–813. 10.1111/j.1469-7793.1999.0803p.x 10420016 PMC2269468

[B21] Latremoliere A. Woolf C. J. (2009). Central sensitization: a generator of pain hypersensitivity by central neural plasticity. *J. Pain* 10 895–926. 10.1016/j.jpain.2009.06.012 19712899 PMC2750819

[B22] Libby P. (2007). Inflammatory mechanisms: the molecular basis of inflammation and disease. *Nutr. Rev.* 65 140–146. 10.1111/j.1753-4887.2007.tb00352.x 18240538

[B23] Ligthart S. Marzi C. Aslibekyan S. Mendelson M. M. Conneely K. N. Tanaka T. (2016). DNA methylation signatures of chronic low-grade inflammation are associated with complex diseases. *Genome Biol.* 17:255. 10.1186/s13059-016-1119-5 27955697 PMC5151130

[B24] Lima F. O. Souza G. R. Verri W. A. Jr. Parada C. A. Ferreira S. H. Cunha F. Q. (2010). Direct blockade of inflammatory hypernociception by peripheral A1 adenosine receptors: involvement of the NO/cGMP/PKG/KATP signaling pathway. *Pain* 151 506–515. 10.1016/j.pain.2010.08.014 20813459

[B25] Malisza K. L. Gregorash L. Turner A. Foniok T. Stroman P. W. Allman A.-A. (2003). Functional MRI involving painful stimulation of the ankle and the effect of physiotherapy joint mobilization. *Magn. Reson. Imaging* 21 489–496. 10.1016/s0730-725x(03)00074-2 12878258

[B26] Mantovani A. Sica A. Sozzani S. Allavena P. Vecchi A. Locati M. (2004). The chemokine system in diverse forms of macrophage activation and polarization. *Trends Immunol.* 25 677–686. 10.1016/j.it.2004.09.015 15530839

[B27] Martins D. F. Bobinski F. Mazzardo-Martins L. Cidral-Filho F. J. Nascimento F. P. Gadotti V. M. (2012). Ankle joint mobilization decreases hypersensitivity by activation of peripheral opioid receptors in a mouse model of postoperative pain. *Pain Med.* 13 1049–1058. 10.1111/j.1526-4637.2012.01438.x 22776137

[B28] Martins D. F. Mazzardo-Martins L. Cidral-Filho F. J. Stramosk J. Santos A. R. S. (2013b). Ankle joint mobilization affects postoperative pain through peripheral and central adenosine A1 receptors. *Phys. Ther.* 93 401–412. 10.2522/ptj.20120226 23086409

[B29] Martins D. F. Mazzardo-Martins L. Cidral-Filho F. J. Gadotti V. M. Santos A. R. S. (2013a). Peripheral and spinal activation of cannabinoid receptors by joint mobilization alleviates postoperative pain in mice. *Neuroscience* 255 110–121. 10.1016/j.neuroscience.2013.09.055 24120553

[B30] Martins D. F. Mazzardo-Martins L. Gadotti V. M. Nascimento F. P. Lima D. A. N. Speckhann B. (2011). Ankle joint mobilization reduces axonotmesis-induced neuropathic pain and glial activation in the spinal cord and enhances nerve regeneration in rats. *Pain* 152 2653–2661. 10.1016/j.pain.2011.08.014 21906878

[B31] McCarson K. E. Fehrenbacher J. C. (2021). Models of inflammation: carrageenan- or Complete Freund’s Adjuvant (CFA)–induced edema and hypersensitivity in the rat. *Curr. Protoc.* 1:e202. 10.1002/cpz1.202 34314105

[B32] McMahon S. B. Malcangio M. (2009). Current challenges in glia-pain biology. *Neuron* 64 46–54. 10.1016/j.neuron.2009.09.033 19840548

[B33] Means T. K. Jones B. W. Schromm A. B. Shurtleff B. A. Smith J. A. Keane J. (2001). Differential effects of a toll-like receptor antagonist on mycobacterium tuberculosis -induced macrophage responses. *J. Immunol.* 166 4074–4082. 10.4049/jimmunol.166.6.4074 11238656

[B34] Medzhitov R. (2008). Origin and physiological roles of inflammation. *Nature* 454 428–435. 10.1038/nature07201 18650913

[B35] Milligan E. D. Watkins L. R. (2009). Pathological and protective roles of glia in chronic pain. *Nat. Rev. Neurosci.* 10 23–36. 10.1038/nrn2533 19096368 PMC2752436

[B36] Mischke J. J. Jayaseelan D. J. Sault J. D. Emerson Kavchak A. J. (2017). The symptomatic and functional effects of manual physical therapy on plantar heel pain: a systematic review. *J. Man. Manip. Ther.* 25 3–10. 10.1080/10669817.2015.1106818 28855787 PMC5539575

[B37] Montilla-García Á. Tejada M. Á. Perazzoli G. Entrena J. M. Portillo-Salido E. Fernández-Segura E. (2017). Grip strength in mice with joint inflammation: a rheumatology function test sensitive to pain and analgesia. *Neuropharmacology* 125 231–242. 10.1016/j.neuropharm.2017.07.029 28760650

[B38] Moss P. Sluka K. Wright A. (2007). The initial effects of knee joint mobilization on osteoarthritic hyperalgesia. *Man. Ther.* 12 109–118. 10.1016/j.math.2006.02.009 16777467

[B39] Mosser D. M. Edwards J. P. (2008). Exploring the full spectrum of macrophage activation. *Nat. Rev. Immunol.* 8 958–969. 10.1038/nri2448 19029990 PMC2724991

[B40] Narita M. Shimamura M. Imai S. Kubota C. Yajima Y. Takagi T. (2008). Role of interleukin-1β and tumor necrosis factor-α-dependent expression of cyclooxygenase-2 mRNA in thermal hyperalgesia induced by chronic inflammation in mice. *Neuroscience* 152 477–486. 10.1016/j.neuroscience.2007.10.039 18262365

[B41] Nielsen M. M. Mortensen A. Sørensen J. K. Simonsen O. Graven-Nielsen T. (2009). Reduction of experimental muscle pain by passive physiological movements. *Man. Ther.* 14 101–109. 10.1016/j.math.2007.12.008 18442947

[B42] Osikowicz M. Mika J. Przewlocka B. (2013). The glutamatergic system as a target for neuropathic pain relief. *Exp. Physiol.* 98 372–384. 10.1113/expphysiol.2012.069922 23002244

[B43] Pan Y. Z. Li D. P. Chen S. R. Pan H. L. (2002). Activation of δ-opioid receptors excites spinally projecting locus coeruleus neurons through inhibition of GABAergic inputs. *J. Neurophysiol.* 88 2675–2683. 10.1152/jn.00298.2002 12424303

[B44] Percie du Sert N. Hurst V. Ahluwalia A. Alam S. Avey M. T. Baker M. (2020). The ARRIVE guidelines 2.0: updated guidelines for reporting animal research. *PLoS Biol.* 18:e3000410. 10.1371/journal.pbio.3000410 32663219 PMC7360023

[B45] Perretti M. Cooper D. Dalli J. Norling L. V. (2017). Immune resolution mechanisms in inflammatory arthritis. *Nat. Rev. Rheumatol.* 13 87–99. 10.1038/nrrheum.2016.193 28053331

[B46] Pettersson L. M. E. Sundler F. Danielsen N. (2002). Expression of orphanin FQ/nociceptin and its receptor in rat peripheral ganglia and spinal cord. *Brain Res.* 945 266–275. 10.1016/s0006-8993(02)02817-2 12126889

[B47] Plein L. M. Rittner H. L. (2018). Opioids and the immune system – friend or foe. *Br. J. Pharmacol.* 175 2717–2725. 10.1111/bph.13750 28213891 PMC6016673

[B48] Radtke C. Vogt P. M. Devor M. Kocsis J. D. (2010). Keratinocytes acting on injured afferents induce extreme neuronal hyperexcitability and chronic pain. *Pain* 148 94–102. 10.1016/j.pain.2009.10.014 19932564

[B49] Raghavendra V. Tanga F. Y. DeLeo J. A. (2004). Complete Freunds adjuvant-induced peripheral inflammation evokes glial activation and proinflammatory cytokine expression in the CNS. *Eur. J. Neurosci.* 20 467–473. 10.1111/j.1460-9568.2004.03514.x 15233755

[B50] Roosterman D. Goerge T. Schneider S. W. Bunnett N. W. Steinhoff M. (2006). Neuronal control of skin function: the skin as a neuroimmunoendocrine organ. *Physiol. Rev.* 86 1309–1379. 10.1152/physrev.00026.2005 17015491

[B51] Rosas R. F. Emer A. A. Batisti A. P. Ludtke D. D. Turnes B. L. Bobinski F. (2018). Far infrared-emitting ceramics decrease Freund’s adjuvant-induced inflammatory hyperalgesia in mice through cytokine modulation and activation of peripheral inhibitory neuroreceptors. *J. Integr. Med.* 16 396–403. 10.1016/j.joim.2018.08.002 30139655

[B52] Salgado A. S. I. Stramosk J. Ludtke D. D. Kuci A. C. C. Salm D. C. Ceci L. A. (2019). Manual therapy reduces pain behavior and oxidative stress in a murine model of complex regional pain syndrome type I. *Brain Sci.* 9:197. 10.3390/brainsci9080197 31405150 PMC6721404

[B53] Samad T. A. Moore K. A. Sapirstein A. Billet S. Allchorne A. Poole S. (2001). Interleukin-1 β-mediated induction of Cox-2 in the CNS contributes to inflammatory pain hypersensitivity. *Nature* 410 471–475. 10.1038/35068566 11260714

[B54] Sauer R.-S. Hackel D. Morschel L. Sahlbach H. Wang Y. Mousa S. A. (2014). Toll like receptor (TLR)-4 as a regulator of peripheral endogenous opioid-mediated analgesia in inflammation. *Mol. Pain* 10:10. 10.1186/1744-8069-10-10 24499354 PMC3922964

[B55] Sluka K. A. Skyba D. A. Radhakrishnan R. Leeper B. J. Wright A. (2006). Joint mobilization reduces hyperalgesia associated with chronic muscle and joint inflammation in rats. *J. Pain* 7 602–607. 10.1016/j.jpain.2006.02.009 16885017

[B56] Sluka K. A. Wright A. (2001). Knee joint mobilization reduces secondary mechanical hyperalgesia induced by capsaicin injection into the ankle joint. *Eur. J. Pain* 5 81–87. 10.1053/eujp.2000.0223 11394925

[B57] Smolen J. S. Aletaha D. McInnes I. B. (2016). Rheumatoid arthritis. *Lancet (London, England)* 388 2023–2038. 10.1016/S0140-6736(16)30173-8 27156434

[B58] Stein C. Machelska H. (2011). Modulation of peripheral sensory neurons by the immune system: implications for pain therapy. *Pharmacol. Rev.* 63 860–881. 10.1124/pr.110.003145 21969325

[B59] Sung Y.-J. Sofoluke N. Nkamany M. Deng S. Xie Y. Greenwood J. (2017). A novel inhibitor of active protein kinase G attenuates chronic inflammatory and osteoarthritic pain. *Pain* 158 822–832. 10.1097/j.pain.0000000000000832 28059868 PMC5402717

[B60] Tapping R. I. Tobias P. S. (2003). Mycobacterial lipoarabinomannan mediates physical interactions between TLR1 and TLR2 to induce signaling. *J. Endotoxin Res.* 9 264–268. 10.1179/096805103225001477 12935358

[B61] Underhill D. M. Ozinsky A. Smith K. D. Aderem A. (1999). Toll-like receptor-2 mediates mycobacteria-induced proinflammatory signaling in macrophages. *Proc. Natl. Acad. Sci. U. S. A.* 96 14459–14463. 10.1073/pnas.96.25.14459 10588727 PMC24458

[B62] Wang S. Lim G. Mao J. Sung B. Yang L. Mao J. (2007). Central glucocorticoid receptors regulate the upregulation of spinal cannabinoid-1 receptors after peripheral nerve injury in rats. *Pain* 131 96–105. 10.1016/j.pain.2006.12.019 17258396

[B63] Wayne W. D. Chad L. C. (2018). *Biostatistics: A Foundation for Analysis in the Health Sciences*, 11th Edn. Hoboken: Willey.

[B64] Weerasekara I. Osmotherly P. Snodgrass S. Marquez J. de Zoete R. Rivett D. A. (2018). Clinical benefits of joint mobilization on ankle sprains: a systematic review and meta-analysis. *Arch. Phys. Med. Rehabil.* 99 1395–1412.e5. 10.1016/j.apmr.2017.07.019 28882509

[B65] Wilson S. R. Thé L. Batia L. M. Beattie K. Katibah G. E. McClain S. P. (2013). The epithelial cell-derived atopic dermatitis cytokine TSLP activates neurons to induce itch. *Cell* 155 285–295. 10.1016/j.cell.2013.08.057 24094650 PMC4041105

[B66] Woolf A. D. Pfleger B. (2003). Burden of major musculoskeletal conditions. *Bull. World Health Organ.* 81 646–656. 14710506 PMC2572542

[B67] Xiang H. C. Lin L. X. Hu X. F. Zhu H. Li H. P. Zhang R. Y. (2019). AMPK activation attenuates inflammatory pain through inhibiting NF-κB activation and IL-1β expression. *J. Neuroinflamm.* 16:34. 10.1186/s12974-019-1411-x 30755236 PMC6373126

[B68] Yadav M. Schorey J. S. (2006). The β-glucan receptor dectin-1 functions together with TLR2 to mediate macrophage activation by mycobacteria. *Blood* 108 3168–3175. 10.1182/blood-2006-05-024406 16825490 PMC1895517

[B69] Yin C. Heit B. (2018). Armed for destruction: formation, function and trafficking of neutrophil granules. *Cell Tissue Res.* 371 455–471. 10.1007/s00441-017-2731-8 29185068

[B70] Zhang R. X. Li A. Liu B. Wang L. Ren K. Zhang H. (2008). IL-1ra alleviates inflammatory hyperalgesia through preventing phosphorylation of NMDA receptor NR-1 subunit in rats. *Pain* 135 232–239. 10.1016/j.pain.2007.05.023 17689191 PMC2323207

[B71] Zhao X.-H. Zhang T. Li Y.-Q. (2015). The up-regulation of spinal Toll-like receptor 4 in rats with inflammatory pain induced by complete Freund’s adjuvant. *Brain Res. Bull.* 111 97–103. 10.1016/j.brainresbull.2015.01.002 25592618

[B72] Zucoloto A. Z. Manchope M. F. Borghi S. M. Dos Santos T. S. Fattori V. Badaro-Garcia S. (2019). Probucol ameliorates complete Freund’s adjuvant-induced Hyperalgesia by targeting peripheral and spinal cord inflammation. *Inflammation* 42 1474–1490. 10.1007/s10753-019-01011-3 31011926

